# Soil Organic Matter Responses to Mangrove Restoration: A Replanting Experience in Northeast Brazil

**DOI:** 10.3390/ijerph18178981

**Published:** 2021-08-26

**Authors:** Laís Coutinho Zayas Jimenez, Hermano Melo Queiroz, Xosé Luis Otero, Gabriel Nuto Nóbrega, Tiago Osório Ferreira

**Affiliations:** 1 Luiz de Queiroz College of Agriculture, University of São Paulo (ESALQ-USP), Av. Pádua Dias 11, Piracicaba 13418-900, SP, Brazil; lais.jimenez@usp.br (L.C.Z.J.); hermanomelo@usp.br (H.M.Q.); 2 CRETUS Institute, Department of Soil Science and Agricultural Chemistry, School of Biology, University of Santiago de Compostela, 15705 Santiago de Compostela, Spain; xl.otero@usc.es; 3 Graduate Program in Earth Sciences (Geochemistry), Department of Geochemistry, Federal Fluminense University, Niterói 24020-141, RJ, Brazil; gabrielnn@id.uff.br

**Keywords:** carbon stock, organomineral interactions, blue carbon, ecosystem restoration

## Abstract

Mangroves are among the most relevant ecosystems in providing ecosystem services because of their capacity to act as sinks for atmospheric carbon. Thus, restoring mangroves is a strategic pathway for mitigating global climate change. Therefore, this study aimed to examine the organic matter dynamics in mangrove soils during restoration processes. Four mangrove soils under different developmental stages along the northeastern Brazilian coast were studied, including a degraded mangrove (DM); recovering mangroves after 3 years (3Y) and 7 years (7Y) of planting; and a mature mangrove (MM). The soil total organic carbon (C_T_) and soil carbon stocks (SCSs) were determined for each area. Additionally, a demineralization procedure was conducted to assess the most complex humidified and recalcitrant fractions of soil organic matter and the fraction participating in organomineral interactions. The particle size distribution was also analyzed. Our results revealed significant differences in the SCS and C_T_ values between the DM, 3Y and 7Y, and the MM, for which there was a tendency to increase in carbon content with increasing vegetative development. However, based on the metrics used to evaluate organic matter interactions with inorganic fractions, such as low rates of carbon enrichment, C recovery, and low C content after hydrofluoric acid (HF) treatment being similar for the DM and the 3Y and 7Y—this indicated that high carbon losses were coinciding with mineral dissolution. These results indicate that the organic carbon dynamics in degraded and newly planted sites depend more on organomineral interactions, both to maintain their previous SCS and increase it, than mature mangroves. Conversely, the MM appeared to have most of the soil organic carbon, as the stabilized organic matter had a complex structure with a high molecular weight and contributed less in the organomineral interactions to the SCS. These results demonstrate the role of initial mangrove vegetation development in trapping fine mineral particles and favoring organomineral interactions. These findings will help elucidate organic accumulation in different replanted mangrove restoration scenarios.

## 1. Introduction

Climate change and global warming have been reported as the most pressing concerns worldwide in recent years [1,2,3,4]. In addition, several environmental issues, such as sea-level rise, extreme weather events, shifting rainfall patterns, and risks for human health and wildlife, are expected to increase in the coming years in response to the climate crisis [1,2,3,4]. Therefore, strategies that can efficiently increase carbon sequestration have a fundamental role in mitigating global warming, especially those that use nature as a tool to restore natural environments [2,5,6].

These “nature-based solutions” [7,8,9] are included in initiatives for the protection, restoration, and sustainable management of ecosystems as they address multiple societal and environmental challenges simultaneously [2,10,11]. Mangrove restoration and protection are essential for achieving sustainable goals, such as climate change mitigation [5,12,13], as mangrove forests are one of the most efficient C sinks. Previous studies have reported that mangroves sequester approximately 13.5 Gt C year^−1^ [14], most of which is captured in their soils (49–98% of the ecosystem C content is stored in the soil) [14,15]. The large C storage and sequester capacity of mangrove forests and other coastal ecosystems (e.g., mangroves) have led to the creation of the term “Blue Carbon sinks” [16,17,18,19].

The stored carbon in mangrove soils exists in living (roots) and non-living biomass (litter and deadwood), as well as in the organic matter incorporated into the soil [15,20,21,22,23]. Soil organic matter (SOM) can remain stored for a millennial scale or be mineralized in the short term (within years or decades) [21,24,25,26]. The high C content in coastal wetland soils occurs because of the specific biogeochemical conditions resulting from the combination of the high primary productivity of the plants and the soil characteristics, such as high salinity, circumneutral pH, mineral interactions, and low availability of oxygen, which compromises organic matter decomposition [18,19].

However, some drivers of the mechanisms of SOM stabilization and protection from decomposition remain poorly understood, especially in coastal wetland ecosystems. Several studies have indicated that the complex molecular structure of SOM is not sufficient to explain its high stability [24] and that organomineral interactions can be recognized as key components for the protection of SOM, thereby preventing decomposition [21,22,23,24]. Accordingly, Fe oxyhydroxides (e.g., ferrihydrite and lepidocrocite) play an important role in preserving organic C in mangrove soils because of the formation of stable complexes by ligand exchange between the organic matter and reactive Fe [23,24,25].

Thus, understanding the mechanisms involved in the C accumulation and stabilization of organic matter in mangrove soils is crucial to comprehensively assess the resilience of these ecosystems in the face of degradation and their development in response to restoration initiatives. Therefore, this study aims to: (i) assess the development of soil carbon stocks (SCSs) in mangrove forests under a revegetation scenario and (ii) identify the role of organomineral interactions on SOM stabilization in mangrove forests under revegetation.

## 2. Materials and Methods

### 2.1. Study Site and Soil Sampling

**Figure 1:**
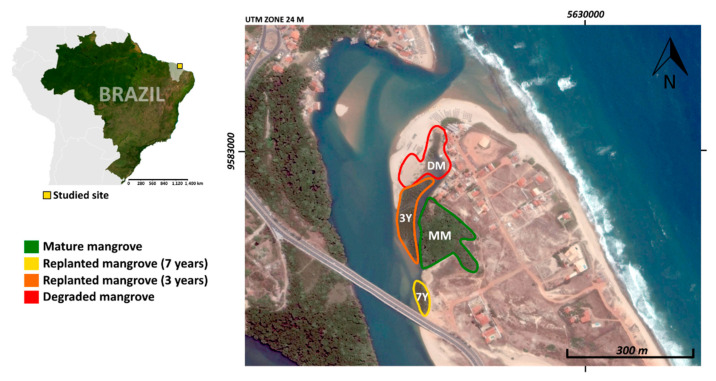
Sampled mangroves at the estuary of Cocó river, northeast Brazil. The location of the planted, mature, and degraded mangroves are highlighted. Satellite image was obtained from Google Earth Pro^TM^, the XY axes represent UTM coordinates. DM: degraded mangrove, 3Y: mangroves with 3 years of planting, 7Y: mangroves with 7 years of planting, MM: mature mangrove forest.

The study site is located in the estuary of the Cocó River in Ceará state, northeast Brazil (Figure 1). The region is characterized by a semiarid climate (BSh, according to Köppen climate classification) with a rainy season (from February to May; ~1000 mm) and an extended dry season from June to January (precipitation ~200 mm), with high evapotranspiration rates (especially during the dry season), and an annual mean temperature of 27 °C [27,28]. In the studied region, the mangroves’ soils are markedly influenced by the “Barreiras” geological group, which is characterized by white to yellow claystones, siltstones, and sandstones, but also to the presence of quartz-dominated dunes [29]. Thus, the soils formed from the “Barreiras” geological group in the Ceará state are mostly highly weathered, kaolinite-dominated, with a minor presence of Fe oxyhydroxides [30]. In this sense, previous studies at the same region reported reactive iron contents of 26 ± 12 mmol kg^−1^, degree of pyritization of 68 ± 9%, and predominance of suboxic conditions [27,28].

Soil cores were collected from four mangrove areas under a restoration chronosequence, based on the age of the planted area during sample collection as follows: a degraded mangrove (DM), where there is a total absence of vegetation; a 3-year-old planted mangrove (3Y), a 7-year-old planted mangrove (7Y), and a mature mangrove (MM), which has been free from degradation for at least 30 years (used as a control). The 3Y planting area covers approximately 3500 m^2^, the 7Y and DM cover approximately 1000 m^2^, and the MM covers 13,000 m^2^. The distances between the areas are approximately 100 m.

Due to the short distance between the studied areas, they have similar positions in the estuary, as well as similar topography, geological context (“Barreiras” formation), tidal regime (mesotidal), hydrodynamic condition, and salinity [31]. The species that predominate in the mature mangroves area are *Avicennia schaueriana* Stapf & Leechman, *Rhizophora mangle* L. and *Laguncularia racemosa* [32]. For restoration (i.e., 3Y and 7Y), degraded areas without vegetation were planted with *Rhizophora mangle* L. propagules [32].

Four undisturbed soil cores (total 16 cores) were collected within 1 m × 1 m in each studied mangrove (DM, 3Y, 7Y, and MM) [33,34]. The cores were collected with 40 cm long polyvinyl chloride tubes (0.5 cm diameter) attached to a stainless-steel sampler for flooded soils during low tide [28]. After soil sampling, the tubes were hermetically sealed and transported vertically to the laboratory under refrigeration (approximately 4 °C). At the laboratory, soil cores were sectioned into 0‒10, 10‒20, 20‒30, and 30‒40 cm depths to obtain subsamples for posterior analyses (i.e., total organic C, particle size, and demineralization to assess the organomineral interactions).

### 2.2. Soil Carbon Contents and SCSs

The total organic carbon (C_T_) content in the soil samples was determined by dry combustion at 1350 °C under pure oxygen using an elemental analyzer (LECO SE-144 DR) after pretreatment with 1 mol L^−1^ HCl to remove carbonates [25]. Meanwhile, the SCSs were quantified using the following: $\mathrm{SCS}=\mathrm{soil}\;\mathrm{bulk}\;\mathrm{density}\times \mathrm{depth}\times C_{T}$ [25].

### 2.3. Particle Size Distribution

The soil particle size distribution was determined using the pipette method [35], after pretreating with hydrogen peroxide (30% solution) to remove the soil organic carbon, followed by mechanical (agitation for 12 h) and chemical dispersions (0.15 mol L^−1^ sodium hexametaphosphate and 1 mol L^−1^ sodium hydroxide).

### 2.4. Soil Demineralization Procedure

**Figure 2:**
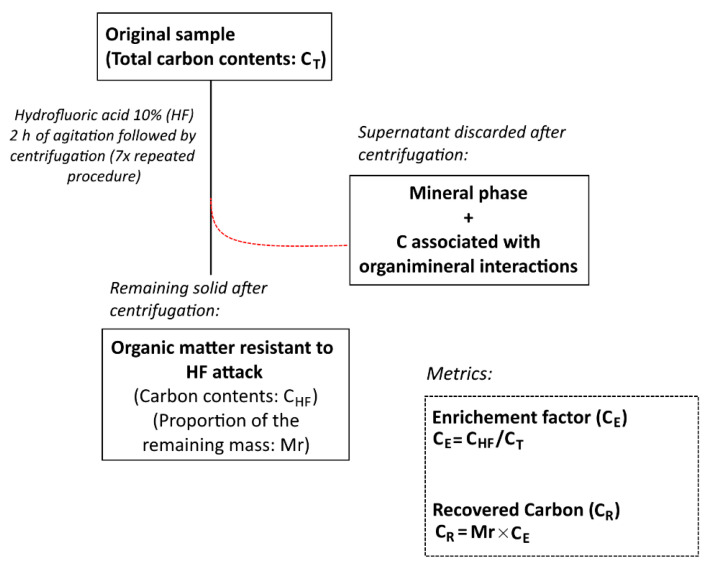
Schematic of the steps involved in the soil demineralization method. The framed texts denote the obtained fractions, unframed texts denote analytical procedures, and the dashed frames contain the used metrics.

A soil demineralization procedure [36] was performed to dissolve the soil mineral phase using hydrofluoric acid (HF, 10% *v*/*v*) in order to concentrate the soil organic fraction (Figure 2) [37]. Specifically, the soil sub-samples were shaken for 2 h with 30 mL of HF acid (10% *v*/*v*), centrifuged, and discarded the supernatant. This procedure was repeated seven times (Figure 2), during which any organic matter attached to the mineral fractions was also discarded [38].

The remaining solid phases were washed three times with deionized water [38], and their masses were determined after oven drying at 60 °C. The ratio of the remaining mass after the HF treatment, as compared with the original mass of the samples, was identified as the “remaining mass” (Mr) [37,39].

The carbon content in the remaining mass of the samples was determined by dry combustion (see Section 2.2). Therefore, the amount of carbon after demineralization with HF is the “Carbon-HF” (C_HF_) (Figure 2).

In general, the C_HF_ contents are higher than the C_T_ contents, as the dissolution of the mineral phase concentrates organic matter. Further, C_HF_ values that are lower than the C_T_ values are related to a loss of carbon associated with the mineral matrix and, thus, indicate an association between the organic fraction and mineral matrix [40].

The C enrichment ratio (C_E_) is the comparison between the carbon contents in the sample before and after HF treatment as follows: $C_{E}=\frac{C_{\mathrm{HF}}}{C_{T}}$ [37,38,39,40]. This parameter reflects the number of times C from the original sample was concentrated after eliminating the mineral fraction.

When multiplying the Mr by the C_E_, we obtain the recovered carbon (C_R_). The C_R_ estimates the fraction of C_T_ that resists the HF treatment [40]. In this sense, the C_R_ is considered the fraction of C_T_ that is not associated with minerals and is, thus, the recalcitrant C (composed of macromolecules with high aromaticity and phenolic groups). In this method, organic C of low molecular weight compounds and particulate organic matter may also be discarded during the flotation and centrifugation procedures [40,41].

The C recovered after HF treatment (C_R_) was calculated using the following [38,40]: $C_{R}\;\left(\%\right)=\mathrm{Mr}\;\left(\%\right)\times \left(C_{\mathrm{HF}}/C_{T}\right)$.

### 2.5. Statistical Analysis

Non-parametric Friedman tests (equivalent to a two-way ANOVA) were performed to assess the differences between soil organic carbon contents, SCS, and soil particle size composition between the studied sites at the 5% significance level, using multiple pair-wise comparisons (software XLSTAT, Version 2014.5.03, New York, NY, USA) [42]. We adopted a non-parametric test because it depends on fewer assumptions and is more robust for environmental data without a normal distribution [42].

## 3. Results and Discussion

**Figure 3:**
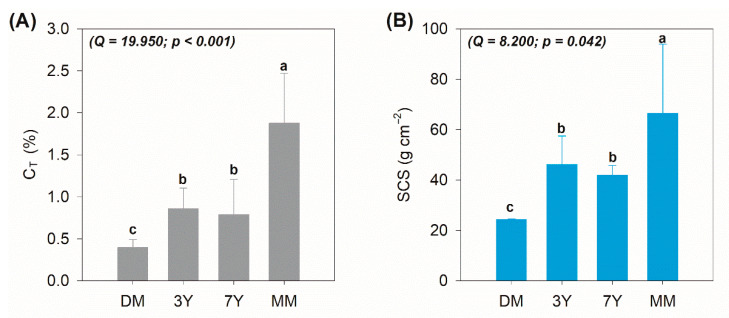
(**A**) Total organic carbon (C_T_) contents and (**B**) soil carbon stocks (SCSs). DM: degraded mangrove, 3Y: mangroves with 3 years of planting, 7Y: mangroves with 7 years of planting, MM: mature mangrove forest. The different lowercase letters on the bars indicate a significant difference between the variables as determined by the Friedman test at a 5% probability level as Q values above the critical Q (7.8147) indicate statistical differences.

The mean C_T_ content of the MM (1.49 ± 0.18%) was significantly higher than those recorded at the planted (3Y: 0.86 ± 0.24%; 7Y: 0.79 ± 0.42%) and degraded area (0.40 ± 0.09%; Figure 3A). There were no significant differences between 3Y and 7Y (Figure 2A), although there was a significant difference between these areas and the degraded mangrove (Figure 3A). These results indicate that carbon contents increased following seedling, which promoted significant increases within 3 and 7 years of planting. These higher carbon contents result from soil carbon inputs from vegetation through root growth and exudates, increased microbial biomass, and plant litter [14,21,26]. The SCS results showed the same patterns as those of the C_T_, in which there were higher SCSs in the MM (66.5 ± 27.4 g cm^−2^) and lower SCSs in the DM (24.3 ± 0.2 g cm^−2^; Figure 3B). No significant SCS differences were observed between the planted areas (3Y: 46.1 ± 11.3 g cm^−2^; 7Y: 41.8 ± 3.9 g cm^−2^; Figure 3B).

**Figure 4:**
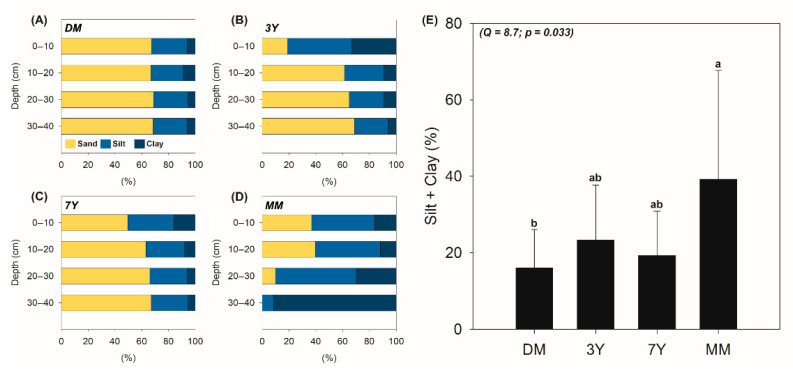
Sand, silt, clay contents for the four studied mangroves, i.e., (**A**) DM: degraded mangrove; (**B**) 3Y: 3-year-old mangroves; (**C**) 7Y: 7-year-old mangroves, and (**D**) MM: mature mangrove forest; (**E**) Statistical analysis of fine particle contents (silt + clay) in each study area. The different lowercase letters on the bars indicate a significant difference between the variables as determined by the Friedman test at a 5% probability level as Q values above the critical Q (7.8147) indicate statistical differences.

In addition to increased input of organic matter, the development of mangrove vegetation also plays an important role in retaining fine mineral particles and increasing sedimentation in the revegetated areas [43,44]. The particle size distribution data support this assumption (Figure 4).

In particular, the particle size contents indicate an increase in fine particles (silt + clay) as vegetation ages (Figure 4E). Statistically higher fine particle contents were found in the MM, followed by the planted (3Y and 7Y) and DM (Figure 4E). The increase in fine particles was especially evident in the soil surface layers of revegetated (i.e., 0–10 cm; Figure 4B‒D). The presence of *Rhizophora mangle* L. higher than 1 m in the revegetated areas likely led to a decrease in the hydrodynamic energy from tides due to its aerial roots and stems and thereby favoring the capture and settlement of fine particles, which would otherwise be easily removed [41,45,46]. This fine sediment trap also contributes to C accumulation by favoring interactions between the organic and mineral phases [47,48], which promote maintaining and increasing the organic C in the mangrove soils [26,41]. Recent studies have reported that soil organic carbon is physically protected by interactions between clay minerals (e.g., kaolinite and smectite) and the functional groups of SOM, which ultimately increases SOM stability against microorganisms and enzymes [41,45,46].

**Figure 5:**
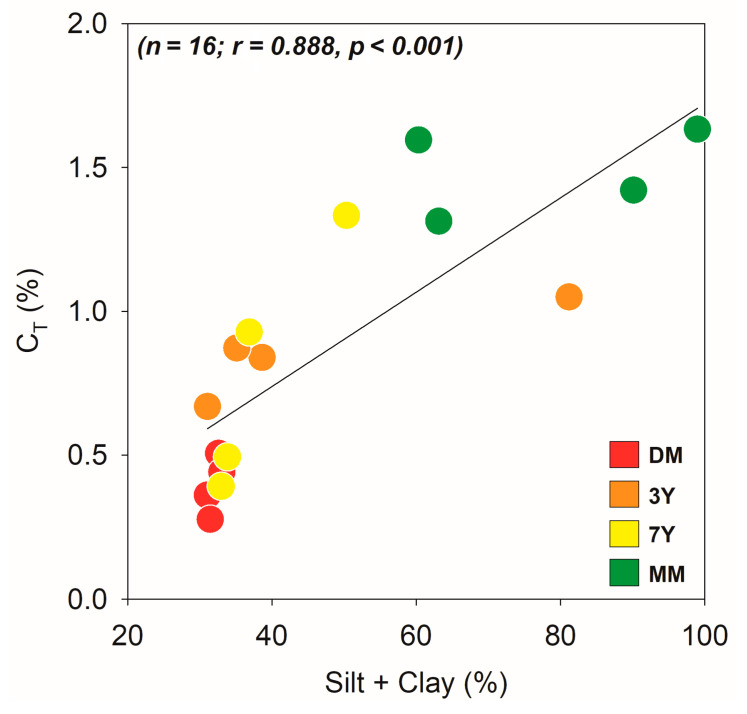
Spearman correlation between fine particles (i.e., silt + clay) and soil total organic carbon content (C_T_) for the studied areas. DM: degraded mangrove, 3Y: mangroves with 3 years of planting, 7Y: mangroves with 7 years of planting, MM: mature mangrove forest, *p* values < 0.05 indicate significant correlation.

In addition, the positive and significant correlation between finer soil particles (silt + clay) and C_T_ (Figure 5) suggests that there is an increased organomineral interaction in mangrove soil with increased fine particles (e.g., clay mineral content). Accordingly, organomineral interactions are expected to increase due to the plantation of mangroves, especially in young planted sites (i.e., 3Y and 7Y), as compared with the DM.

**Figure 6:**
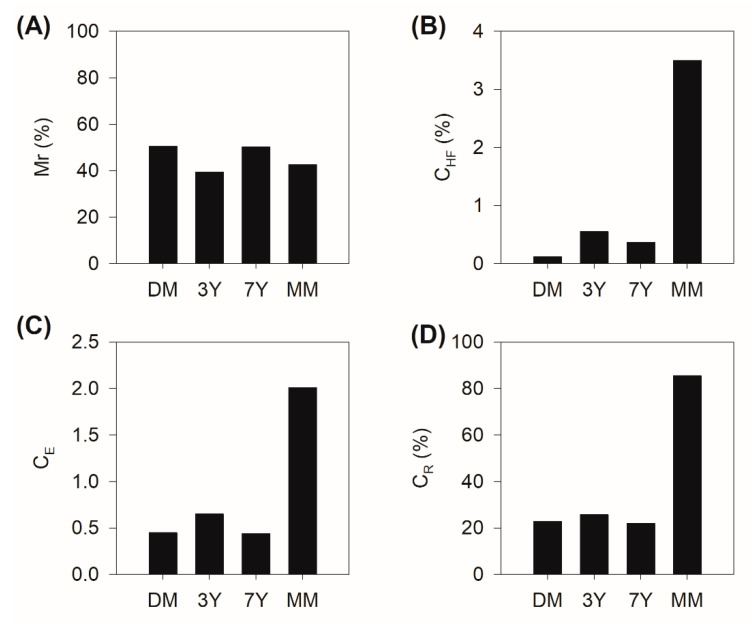
(**A**) Remaining mass (Mr); (**B**) Carbon contents after hydrofluoric acid (HF) treatment (C_HF_); (**C**) Carbon enrichment (C_E_), and (**D**) Carbon recovery (C_R_) results of the demineralization procedure for the degraded mangrove (DM), replanted mangroves (3Y and 7Y), and mature mangrove (MM).

The particle size distribution also affects the amount of remaining mass after HF treatment (Mr). The Mr values found for DM, 3Y, and 7Y (50.5%, 39.4%, and 50.3%, respectively; Figure 6A) are high, which is probably related to the predominance of sand particles in these areas [43,44]. In the mangrove soils of northeast Brazil, the sand fraction is mainly composed of quartz, which resists HF treatment [40,43]. Previous studies have reported a lower mass loss after HF treatment in sand-rich soils than in clayey soils [40,43,49,50]. Meanwhile, in the MM, although there is a predominance of fine particles (Figure 4), the Mr values were also considerably high (39%; Figure 4A). Unlike in the other areas, this resistance to HF treatment may be related to the presence of organic matter with a high molecular weight, as observed in previous studies, not the particle size distribution [37,38,39]. The loss of carbon due to HF treatment in the MM was negligible (Figure 6B), indicating that the organomineral interactions exhibit less influence, and there is less participation of low molecular weight organic matter and particulate organic matter, which can also be eliminated during the flotation and centrifugation procedures [38].

Accordingly, the C_E_ values were lower than 1 in the DM, 3Y, and 7Y sites (Figure 6C), indicating that the C_HF_ contents were lower than the C_T_ content (Figure 6B). The significant losses of C during the dissolution of minerals performed with HF suggest that, in these three areas, there is a high contribution of organic matter associated with the mineral matrix [37,38,39,40]. Thus, organomineral interactions may be the main mechanisms of organic matter protection in the DM, 3Y, and 7Y. Previous studies have reported that C_E_ values of less than 1 are related to the predominance of organic matter associated with mineral phases [37,38,39]. Thus, the CE value of less than 1 (2.01, Figure 6B) in the MM indicates a lower contribution of organic matter associated with the mineral matrix and a greater contribution of high molecular weight organic matter [49,50]. These results corroborate the higher degree of stability and lower lability of organic matter in the MM, as previously suggested.

The C_R_ results also demonstrate a higher contribution of organic matter related to mineral phases (i.e., clay minerals) in the DM, 3Y, and 7Y, as compared with the MM (Figure 6D). In addition, relative amounts of organic matter resistant to HF treatment (expressed as C_R_) increased from the DM to the MM (Figure 6C). Specifically, in the DM, 3Y, and 7Y, the C_R_ values represented approximately 20% of the total carbon (average: 22.8%, 25.7, and 22.8., respectively), whereas in the MM, the values were significantly higher (average: 85.5%; Figure 6D). The significantly higher C_R_ values in the MM, in addition to negligible C losses upon the dissolution of silicates and oxides, indicate a greater contribution of an intrinsically resistant organic matter in this area (i.e., matter with a high molecular weight, more complex structure, composed of complex macromolecules and associated with their micelles) [39,40,43]. In particular, higher recalcitrance is expected in the MM because macromolecules with high aromaticity and phenolic groups accumulate over time in soils in response to continuous organic matter input [24,43,49].

The high contribution of organomineral interactions in the DM and the replanted areas indicates the relevance of these associations in the resilience of mangrove carbon when degraded and the relevance of this association at the initial stages of carbon stock establishment. Moreover, recent studies have shown that newly added organic matter is associated with pre-existing organomineral fractions [48]. Thus, organomineral associations in recently planted mangroves may be significant for carbon stock recovery [48]. However, further studies are required to investigate the dependence of SCS resilience on the molecular structure of organic matter in degraded mangroves and areas under recent planting. In this sense, new studies focused on physicochemical (redox potential and pH) changes after revegetation are essential to assess the effects of these changes on the reestablishment of carbon stock. For example, oxygen diffusion to plant roots, oxidizing the rhizosphere [51] oxidize soluble Fe(II), leading to the formation of oxyhydroxides that may act on soil organic matter stabilization in mangrove soils [52,53,54,55].

## 4. Conclusions

Our data revealed the key role of organomineral interactions in mangrove soils during the early stages of mangrove vegetation and highlighted the importance of vegetation development in trapping fine particles and promoting organomineral interactions as an essential pathway for protecting SOM. Conversely, the maintenance of carbon stocks in mature mangroves depends on highly complex and recalcitrant SOM produced over time.

These findings help elucidate how the organic accumulation process occurs under different replanted mangrove restoration scenarios. These results bring novel knowledge for strategies that can efficiently enhance carbon sequestration through mangrove forest restoration.

In addition, the restoration of degraded areas does not reflect an immediate increase in the organic C content in the soil, but it is consistent with the delay that the edaphic processes imply.

This study shows that understanding the mechanisms of organic matter stabilization is pivotal for future studies focused on preserving soil carbon pools in mangrove forests.
